# Inferior Vena Cava Placement of a Transhepatic Tunneled Dialysis Catheter in a Patient with Atypical Hepatic Venous Anatomy: A Case Report

**DOI:** 10.7759/cureus.69365

**Published:** 2024-09-13

**Authors:** Hieu M Vo, Raeeha Syeda, Mohammad Ali

**Affiliations:** 1 Department of Interventional Radiology, William Carey University College of Osteopathic Medicine, Hattiesburg, USA; 2 Department of Interventional Radiology, Mississippi Baptist Medical Center, Jackson, USA

**Keywords:** atypical hepatic venous anatomy, case report, end-stage renal disease (esrd), hemodialysis access, transhepatic dialysis catheter

## Abstract

A transhepatic hemodialysis (HD) catheter serves as a final option for obtaining HD vascular access in patients whose conventional access sites, including internal jugular veins, external jugular veins, and femoral veins, are no longer viable. This could be due to intravascular thrombosis or central venous stenosis, among others. The ideal catheter tip position in transhepatic tunneled dialysis catheter is the right atrium for optimal blood flow necessary for dialysis. The report presents a case of a 46-year-old female, in whom the traditional vascular access sites for dialysis were not achievable and, thus, required the use of the hepatic access route. However, her case was further complicated due to the unique hepatic vascular anatomy, causing repeated retraction of the catheter tip from the right atrium to the inferior vena cava (IVC) and hepatic vein. This was circumvented by the atypical placement of the catheter tip down to the suprarenal IVC, deep enough to lodge the catheter in place with adequate flow for successful HD.

## Introduction

Hemodialysis (HD) is the most frequent and common mode of kidney replacement for patients with chronic renal failure, and well-functioning vascular access is essential for successful dialysis therapy [1]. The most commonly used vascular access routes are arteriovenous fistulas, arteriovenous grafts, and HD catheters, each with advantages and disadvantages [1].

In addition to the options mentioned above for vascular access, there are also various options for vascular access sites for dialysis [1]. The following veins are preferred in the order presented: internal jugular veins, external jugular veins, femoral veins, and subclavian veins [1]. If the common access sites are no longer accessible, the next option would be the translumbar inferior vena cava (IVC) approach [2]. If the option of translumbar IVC has also been exhausted, a transhepatic approach would be the next option [2].

In the transhepatic approach, the middle and right hepatic veins are usually accessed laterally using a percutaneous needle puncture at the eighth intercostal space and mid-axillary line [1,2]. The catheter tip should be placed in the right atrium for optimal flow and toxin clearance from adequately mixed blood [3]. It also reduces possible malposition and malfunction of the catheter due to thrombosis and contact with the vessel wall [4]. A retrospective study analyzed the safety and functionality of transhepatic tunneled hemodialysis catheters (TDC) placed in 38 participants. This study concluded that the transhepatic route of placing a tunneled catheter was a safe alternative with low risks when other vascular options were no longer viable [5].

However, just like the other options, the transhepatic approach for dialysis has its drawbacks [1]. This method has shown increased cases of thrombosis and dislodgement compared to the translumbar approach, with many authors describing the short course for the catheter through the hepatic vein and into the right atrium [1]. Rarely, hematomas and a single case of death from hemorrhagic shock have also been reported due to the transhepatic approach [1].

## Case presentation

A 46-year-old female with a past medical history of end-stage renal disease (ESRD) on HD, systemic lupus erythematosus, and rheumatoid arthritis was transferred from an outside hospital specifically for transhepatic TDC due to blockage of left upper extremity HD access. Temporary catheter placement on the right groin was attempted, but it was unusable. Her last dialysis was three days before. Even though the translumbar IVC approach would be the next recommended option before the transhepatic approach, it was not ideal due to the patient's history of stenoses in the IVC and multiple angioplasties being needed at her IVC filter level.

The patient appeared chronically ill but not in acute distress. Her vitals were as follows: blood pressure was 166/98 mmHg, heart rate was 84 beats/minute, temperature was 97.7°F (36.5°C), respiratory rate was 18 breaths/minute, and SpO_2_ was 98%. The patient reported swelling of the left upper extremity before the recent dialysis. Given the history of extensive stenoses affecting the chest, neck, lower extremities, and the history of IVC stenosis precluding the translumbar approach, transhepatic HD access was chosen.

**Figure 1:**
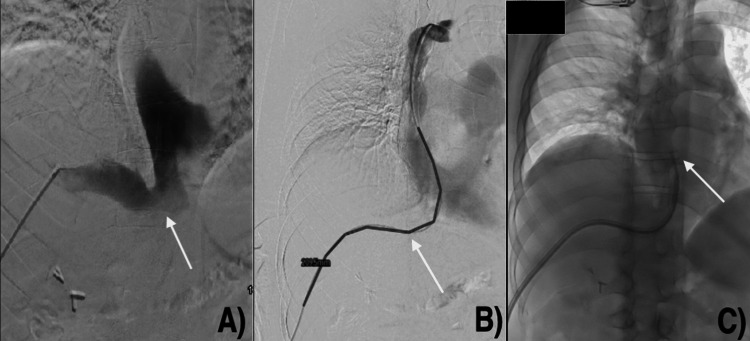
First attempt at transhepatic TDC placement. (A) Right hepatic venogram demonstrated that the right hepatic vein drains into the IVC at an almost right angle (arrow). (B) Another venogram of the SVC with the catheter following the unique venous anatomy (arrow). (C) Catheter tip ended at the right atrium (arrow) TDC: tunneled hemodialysis catheter; IVC: inferior vena cava; SVC: superior vena cava

Before the procedure, CT of the abdomen with and without contrast (triple-phase liver) was performed to evaluate the liver's anatomy and vasculature. The CT showed an incidental right renal mass and mild intrahepatic and extrahepatic biliary dilatation. The patient underwent placement of a 28 cm tip-to-cuff transhepatic HD catheter (Covidien Palindrome, Medtronic, Minneapolis, MN) under ultrasound and fluoroscopic guidance with venography (Figure 1). This demonstrated the unique vascular anatomy, where the right hepatic vein entered the IVC at almost a right angle.

**Figure 2:**
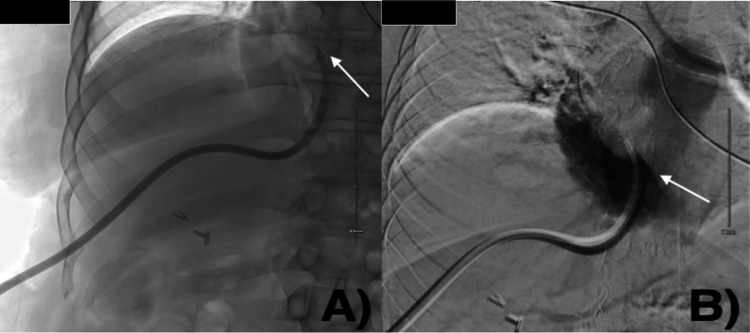
(A) Fluoroscopy showing catheter tip retracted into the IVC (arrow). (B) A venogram was obtained demonstrating contrast accumulating near the junction of IVC and left hepatic vein (arrow) IVC: inferior vena cava

**Figure 3:**
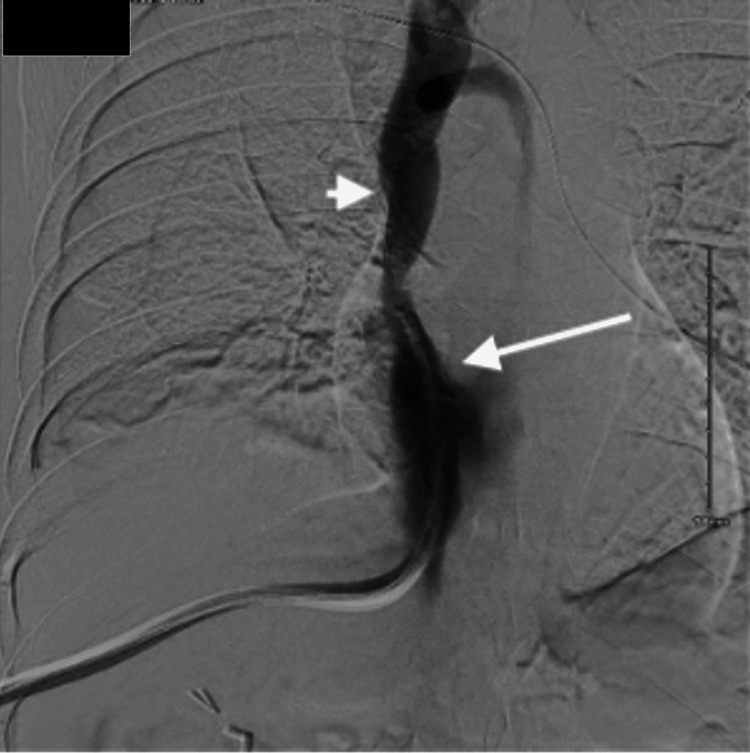
Venogram with contrast, showing the final catheter position on the second attempt in the right atrium (arrow) with the superior vena cava opacified (arrowhead)

Subsequent HD indicated suboptimal flow, with an average blood flow rate of 250 mL/minute. The patient was sent to interventional radiology for readjustment. The transhepatic TDC was retracted into the IVC (Figure 2). The catheter was successfully repositioned into the mid-right atrium (Figure 3). The next attempt at HD failed.

**Figure 4:**
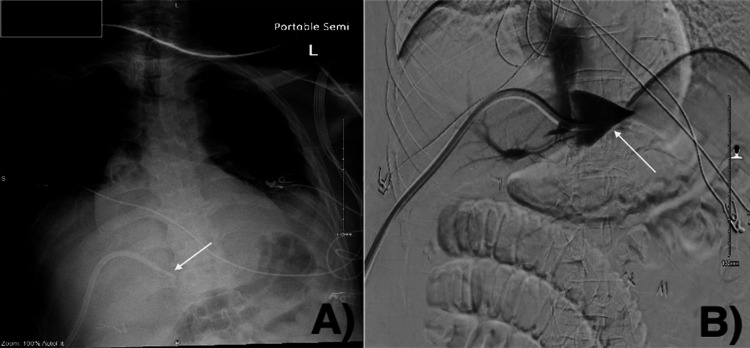
(A) CXR showing the transhepatic TDC in the RUQ (arrow). (B) Venogram showing contrast opacifying the left hepatic vein (arrow) TDC: tunneled hemodialysis catheter; RUQ: right upper quadrant

CXR done the next day indicated that the transhepatic TDC was retracted within the right upper quadrant of the abdomen (Figure 4A). Fluoroscopy showed repeated retraction of the transhepatic TDC into the left hepatic vein per venogram (Figure 4B). A possible explanation includes the 90° angle at which the right hepatic joins the IVC coupled with breathing movement.

**Figure 5:**
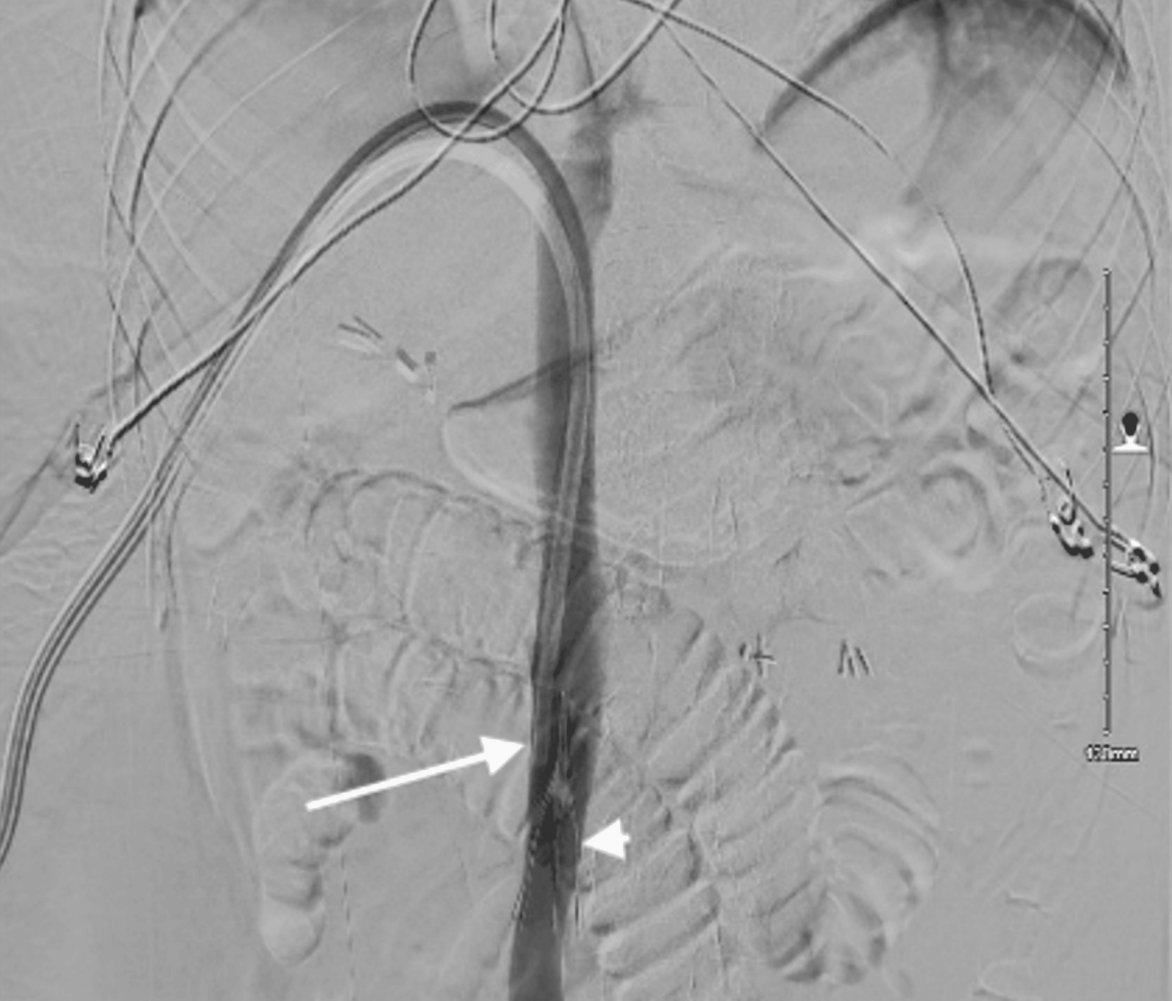
Venogram showing the catheter tip ending in the IVC (arrow) just above the IVC filter (arrowhead) IVC: inferior vena cava

**Figure 6:**
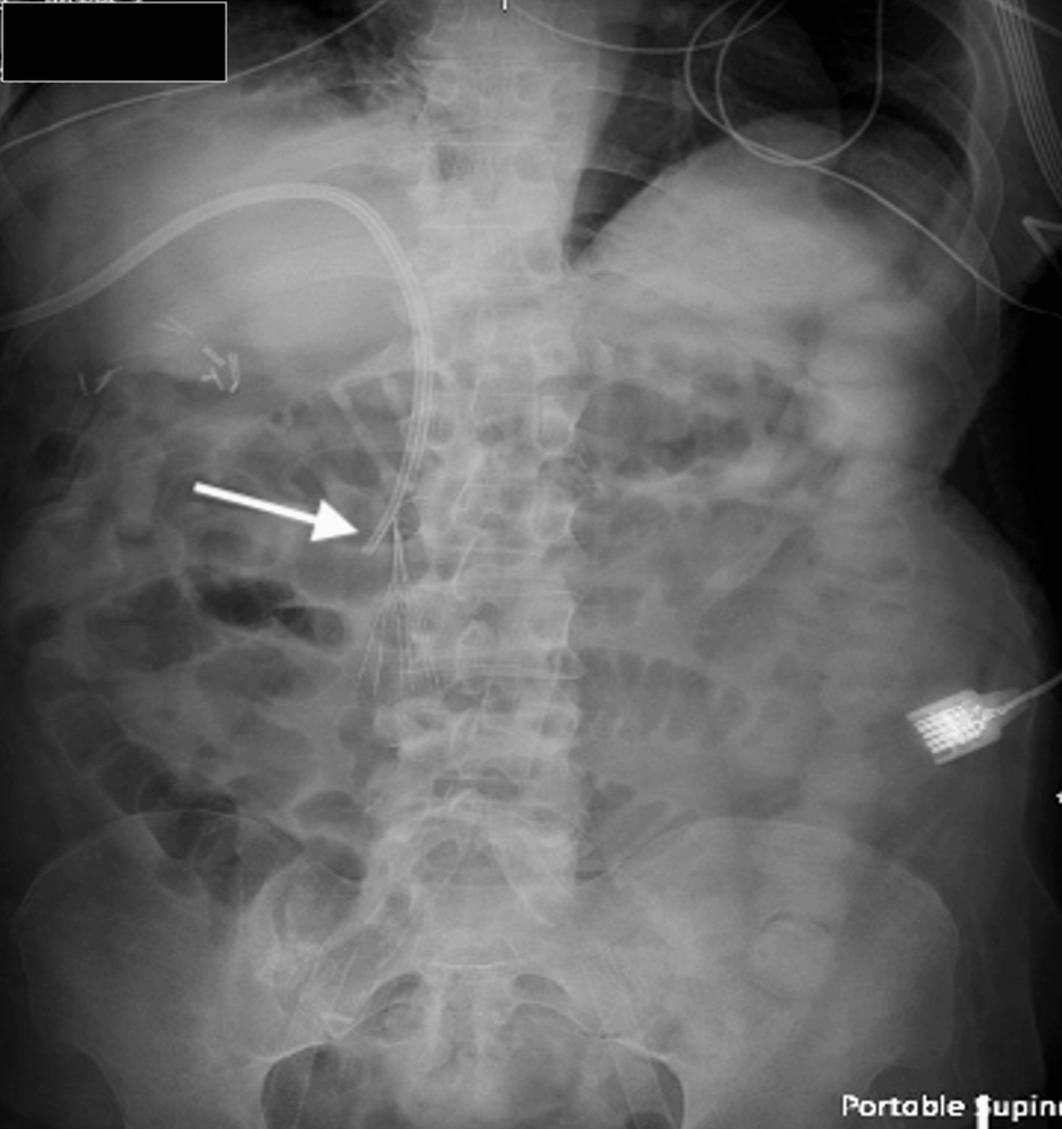
KUB the day after the transhepatic TDC exchange demonstrated the stable position of the catheter (arrow) KUB: kidney, ureter, and bladder; TDC: tunneled hemodialysis catheter

The 28 cm tip-to-cuff transhepatic TDC was exchanged for a 33 cm tip-to-cuff TDC. The catheter tip was positioned deep in the IVC, just above the IVC filter (Figure 5). A cavogram demonstrates a widely patent IVC. The patient successfully underwent HD. A kidney, ureter, and bladder X-ray was performed, which showed a stable position of the catheter at the suprarenal IVC just above the IVC filter (Figure 6).

**Figure 7:**
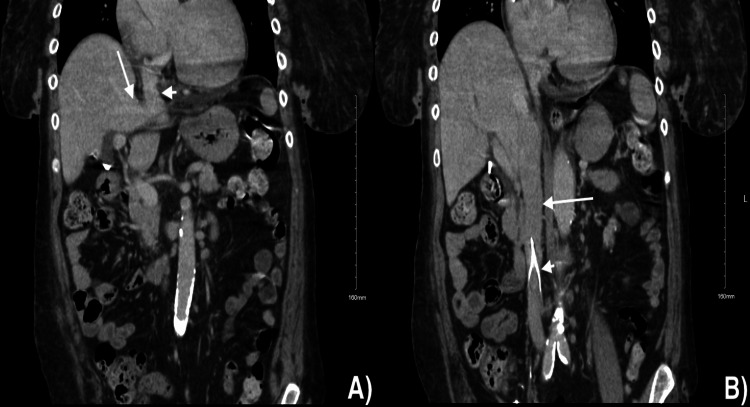
Coronal views of the preprocedural CT of the abdomen. (A) Right hepatic vein (arrow) going into the IVC (arrowhead) at a nearly right angle. (B) IVC (arrow) is patent with the IVC filter (arrowhead) in place IVC: inferior vena cava

In retrospect, the CT of the abdomen before the procedure indicates the nearly right angle between the right hepatic vein and IVC (Figure 7). It also shows that the IVC and the IVC filter were patent, allowing for possible translumbar TDC placement instead of transhepatic placement.

It was decided that the patient would be placed on peritoneal dialysis as a backup should any complications with her transhepatic tunneled catheter arise.

## Discussion

Vascular access is the cornerstone of dialysis treatment in patients with ESRD [2]. Translumbar and transhepatic approaches are the last resort if conventional access sites are no longer viable. For most access sites, the HD catheter is ideally placed in the right atrium or cavoatrial junction for optimal flow and clearance and to reduce the risk of catheter malposition or malfunctioning.

The present case demonstrates the challenge of placing transhepatic TDC due to the unique hepatic venous system, with the right hepatic vein entering the IVC at a nearly right angle, which causes repeated retraction of the catheter tip down to the IVC and hepatic vein, leading to suboptimal HD. It also shows the feasibility of positioning the HD catheter in the suprarenal IVC with adequate blood flow for HD and stability, even if being superjacent to the IVC filter. For this patient, transhepatic TDC is likely the last viable access and should not be removed unless necessary, such as in cases of hemodynamic instability from sepsis. If it is pulled, replacement of the transhepatic catheter may not be possible. Long-term follow-up is needed to monitor for possible complications that may arise from this atypical placement. The patient will also have peritoneal dialysis placed as a backup in case any complications arise.

Transhepatic HD catheters are associated with a high risk of dislodgement and migration, infection, and thrombosis [2]. In retrospect, we think that the translumbar approach would have been possible and worth trying. There is a stenosis at the level of the filter as it was difficult to pass the wire into the IVC below the filter. An intravascular ultrasound would be able to confirm the stenosis.

## Conclusions

This case report highlights a unique challenge of transhepatic HD catheter placement due to unique hepatic venous anatomy, specifically the right hepatic vein entering the IVC at a right angle, resulting in repeated catheter retraction and suboptimal HD. By repositioning the catheter tip in the suprarenal IVC, stability and adequate HD were achieved. This unique approach necessitates peritoneal dialysis as a backup as well as monitoring for potential complications.
